# Soluble Major Histocompatibility Complex Class I-Related Chain B Molecules Are Increased and Correlate With Clinical Outcomes During Rhinovirus Infection in Healthy Subjects

**DOI:** 10.1378/chest.13-2247

**Published:** 2014-02-20

**Authors:** Aurica G. Telcian, Mihnea T. Zdrenghea, Gaetano Caramori, Vasile Laza-Stanca, Simon D. Message, Tatiana Kebadze, Onn M. Kon, Veronika Groh, Alberto Papi, Sebastian L. Johnston, Patrick Mallia, Luminita A. Stanciu

**Affiliations:** From the Airways Disease Infection Section (Drs Telcian, Laza-Stanca, Message, Kebadze, Mallia, and Stanciu and Prof Johnston), National Heart & Lung Institute (NHLI), and Centre for Respiratory Infection, Imperial College London; Medical Research Council; and Asthma UK Centre in Allergic Mechanisms of Asthma, London, England; The Oncology Institute “Prof. Dr. Ion Chiricuţă” Cluj-Napoca and Iuliu Haţieganu University of Medicine and Pharmacy Cluj-Napoca (Drs Zdrenghea and Stanciu), Cluj-Napoca, Romania; Centro Interdipartimentale per lo Studio delle Malattie Infiammatorie delle Vie Aeree e Patologie Fumo-correlate (CEMICEF) (Dr Caramori and Prof Papi), Sezione di Malattie dell’Apparato Respiratorio, Università degli Studi di Ferrara, Ferrara, Italy; Imperial College Healthcare NHS Trust (Drs Message, Kon, and Mallia and Prof Johnston), London, England; and Fred Hutchinson Cancer Research Center (Dr Groh), Seattle, WA.

## Abstract

**BACKGROUND:**

Surface major histocompatibility complex class I-related chain (MIC) A and B molecules are increased by IL-15 and have a role in the activation of natural killer group 2 member D-positive natural killer and CD8 T cells. MICA and MICB also exist in soluble forms (sMICA and sMICB). Rhinoviruses (RVs) are the major cause of asthma exacerbations, and IL-15 levels are decreased in the airways of subjects with asthma. The role of MIC molecules in immune responses in the lung has not been studied. Here, we determine the relationship between MICA and MICB and RV infection in vitro in respiratory epithelial cells and in vivo in healthy subjects and subjects with asthma.

**METHODS:**

Surface MICA and MICB, as well as sMICA and sMICB, in respiratory epithelial cells were measured in vitro in response to RV infection and exposure to IL-15. Levels of sMICA and sMICB in serum, sputum, and BAL were measured and correlated with blood and bronchoalveolar immune cells in healthy subjects and subjects with asthma before and during RV infection.

**RESULTS:**

RV increased MICA and MICB in vitro in epithelial cells. Exogenous IL-15 upregulated sMICB levels in RV-infected epithelial cells. Levels of sMICB molecules in serum were increased in healthy subjects compared with subjects with stable asthma. Following RV infection, airway levels of sMIC are upregulated, and there are positive correlations between sputum MICB levels and the percentage of bronchoalveolar natural killer cells in healthy subjects but not subjects with asthma.

**CONCLUSIONS:**

RV infection induces MIC molecules in respiratory epithelial cells in vitro and in vivo. Induction of MICB molecules is impaired in subjects with asthma, suggesting these molecules may have a role in the antiviral immune response to RV infections.

Respiratory virus infections, particularly by rhinoviruses (RVs), are a major cause of morbidity and mortality and the most common triggers of asthma exacerbations. The mechanisms of these virus-induced asthmatic exacerbations remain poorly understood. Respiratory viruses primarily enter and replicate in epithelial cells in the respiratory tract and epithelial cells play a key role in initiating and modulating immune responses to infection. Epithelial cells express surface molecules such as the major histocompatibility complex (MHC) class I molecules that could activate cytotoxic T cells.^1,2^ New molecules related to MHC class I molecules, the MHC class I-related chain (MIC) A and B molecules, have been reported to be upregulated on stressed and/or rapidly proliferating cells, which includes pathogen-infected cells.^3^ Surface MIC molecules bind the nonspecific activatory receptor natural killer (NK) group 2 member D (NKG2D), leading to activation of NK and CD8 T cells and, therefore, may be important in promoting immune responses to virus infections.^4‐6^

MICA and MICB are also released in biologic fluids as soluble MIC molecules (sMICA and sMICB).^7,8^ However, even in data reports, levels of MICA and MICB were measured together by using an antibody recognizing both MICA/MICB (anti-MICA/B monoclonal antibodies clones 6D4).^9,10^ Individual antibodies to identify sMICA and sMICB molecules individually recently became available.

Innate cytokines induced by viruses, type 1 interferons and IL-15, modulate each other and have an important role in antiviral immune responses. In addition, they increase surface MICA/MICB in dendritic cells, macrophages, and intestinal epithelial cells.^11‐14^ Subjects with asthma have increased susceptibility to RV infections, and a deficient type 1 antiviral immune response has been suggested as one of the mechanisms involved in RV-induced asthma exacerbations.^15‐19^ Epithelial and BAL cells exposed in vitro to RV demonstrated impaired production of innate interferons in atopic subjects with asthma, and these deficiencies were related to severity of RV-induced asthma exacerbations in vivo.^17,18^ In addition, both BAL IL-15 levels and induction of IL-15 in vitro by RV in alveolar macrophages are deficient in subjects with mild asthma, and these deficiencies are related to virus load and disease severity in vivo during a subsequent experimental RV infection.^19^

We previously reported that infection of respiratory epithelial cells in vitro with respiratory syncytial virus increased IL-15 and surface MICA and sMICA levels.^2^ However, the role of RV and IL-15 in the modulation of surface MICA and MICB and sMICA and sMICB in respiratory epithelial cells and the role of sMICA and sMICB in RV infection in healthy subjects and in virus-induced asthma exacerbations are not known. We investigated in vitro the cell surface and soluble levels of MICA and MICB in RV-infected epithelial cells and investigated the role of exogenous IL-15 on MICA and MICB levels in RV-infected epithelial cells. Finally, we correlated sMICA and sMICB levels in biologic fluids (serum, sputum, and BAL) to the immune responses and clinical illness severity in vivo in healthy subjects and subjects with asthma exposed to an experimental RV infection.

We report here that in vitro there is increased production of sMICA and sMICB molecules in RV-infected epithelial cells and that exogenous IL-15 increased surface MICB and sMICB levels. In vivo levels of serum sMICB are decreased in subjects with asthma as compared with healthy subjects, and sMICB molecules in the airways correlated to the percentage of NK cells in BAL and sMICB levels in serum correlated to upper and lower respiratory symptoms during RV infection in healthy subjects.

## Materials and Methods

RV16 (major group RVs) and RV1B (minor group RV) stocks were prepared and their identities confirmed by neutralization using serotype-specific antibodies (American Type Culture Collection). To assess the specificity of live RV-mediated responses, we used ultraviolet (UV)-inactivated and filtered RV (produced by passing RV stocks through a 30-kDa membrane; EMD Millipore Corp).^1^ Bronchial epithelial cells (BEAS-2B) (European Collection of Cell Cultures) were cultured and infected with RV as previously described.^1,20^ Semiconfluent cell monolayers were exposed to RV at different multiplicity of infections (MOIs) (infectious units per cell) or inactivated RV for 1 h with gentle shaking, the virus was washed, and fresh medium ± IL-15 (5 ng/mL) (R&D Systems, Inc) was added. Time zero (0 h) was considered the moment when virus was removed. Cells and supernatants were harvested at different time points 24, 48, 72, and 96 h.

Flow cytometry was used to determine surface levels of MICA and MICB. Epithelial cells were harvested at different time points and processed as previously described.^21^ Surface MICA and MICB were detected on cells by direct staining using mouse anti-human antibodies: MICA (phycoerythrin) and MICB (allophycocyanin) (all from R&D Systems, Inc).^2^ At least 10,000 events were acquired using a BD LSR flow cytometer and CellQuest software (BD Biosciences). Results were expressed as mean fluorescent intensity (MFI) after subtracting the MFI of the control cells stained with the appropriate isotype control antibodies. Stimulation experiments were expressed as fold increase of MFI of experimental condition over medium-treated cells of at least three separate experiments (three to six experiments).

The clinical details, including allergy testing and lung function, sampling and analysis of the experimental infection model, have already been described in detail.^16^ The study recruited 15 nonatopic healthy control subjects and 10 atopic, inhaled steroid-naive subjects with asthma; all were nonsmokers and negative for RV16 serum-neutralizing antibodies. The asthmatic group was required to have a provocative concentration causing a 20% drop in FEV_1_ < 8 mg/mL on histamine challenge. In addition, a provocative concentration causing a 10% drop in FEV_1_ was determined in both patients with asthma and normal subjects. Their demographic and clinical features are described in detail elsewhere.^16^ Ethics approval (no. 99/BA/345) was obtained from St. Mary’s Local Research Ethics Committee (London, England). All study participants gave written informed consent. Serum, sputum, and BAL were collected at baseline approximately 2 weeks prior to RV infection as previously described.^16^ Two weeks later, the same subjects were experimentally infected with RV16, and MICA and MICB levels were measured in serum (at day 3, 4, 7, and 6 weeks after infection), sputum (at day 3, 7, and 6 weeks after infection), and BAL fluid (at day 4 and 6 weeks after infection). Virus load was measured using quantitative polymerase chain reaction as described.^16^ Symptom severity during the 2 weeks postinfection was derived using daily diary cards to score upper respiratory (cold) and lower respiratory (chest) symptoms as described.^16^ Peripheral blood cell counts were measured in the Clinical Biochemistry and Hematology Laboratories of St. Mary’s Hospital, Imperial College Healthcare NHS Trust.

BAL was collected in a single plastic chamber and transferred immediately to polypropylene tubes on ice for transport to the laboratory. The BAL cell pellet was used for cytospin preparations for differential cell counting as described.^16^ Surface staining of fresh blood and BAL cells with conjugated antibodies and analysis by three-color and four-color flow cytometry was performed as previously described^16^ to assess the percentage and total cell numbers of lymphocytes, T cells (CD3^+^), CD4 T cells (CD3^+^CD4^+^CD8^−^), CD8 T cells (CD3^+^CD8^+^CD4^−^), NK T cells (CD3^+^CD16/56^+^), and NK cells (CD3^−^CD16/56^+^). Analysis was performed on at least 10,000 lymphocyte events using CellQuest (BD Biosciences) and Winlist (Verity Software House) software.

MICA and MICB levels were measured in cell supernatants and biologic fluids using paired antibodies and standards (DuoSet; R&D Systems, Inc). Assay sensitivities were 15.62 pg/mL (MICA) and 39.06 pg/mL (MICB). Levels of IL-15 were measured in cell supernatants by enzyme-linked immunosorbent assay using commercially available paired antibodies (R&D Systems, Inc) (sensitivity 0.39 pg/mL).^2,19^

### Statistical Analysis

The results were analyzed using GraphPad Prism (GraphPad Software). Results of in vitro experiments were expressed as mean ± SEM. Analysis of variance, followed where appropriate by paired Student *t* test, was used for the comparisons between different experimental conditions. Correlations were determined by using Spearman rank correlation. *P* values < .05 were considered statistically significant.

## Results

### RV Infection Increases MIC

Surface MICA levels on epithelial cells were increased by RV1B at 24 h, and this was replication-dependent (UV-inactivated RV1B did not increase surface MICA) and dose-dependent (MFI was 7.9 ± 1.6 for medium-treated cells, 11.1 ± 0.9 for UV-RV1B-treated cells, 40.7 ± 13.3 for RV1B MOI of 1, and 46.3 ± 13.3 for RV1B MOI of 3; *P* < .05 medium vs RV1B MOI of 3, and *P* < .05 RV1B MOI of 3 vs MOI of 1, data not shown). Surface MICB levels on epithelial cells were not found to be upregulated by RV infection at 24 h (MFI was 88.9 ± 25.1 for medium-treated cells, 16 ± 10.1 for RV1B MOI of 1, and 5.1 ± 4.1 for RV1B MOI of 3, data not shown).

**Figure 1:**
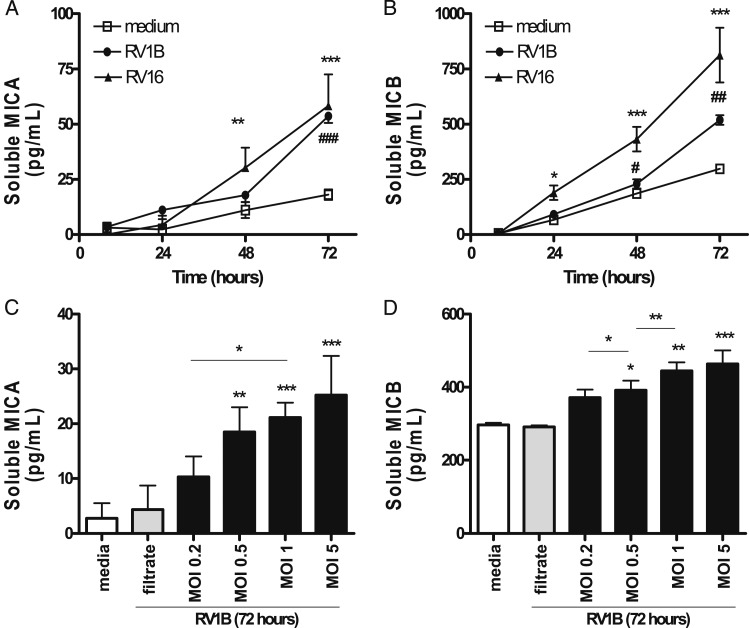
– RV infection increases in vitro levels of soluble MICA and MICB in bronchial respiratory epithelial cells. BEAS-2B respiratory epithelial cells were infected with RV1B and RV16 at different concentrations, or cultured with medium alone or filtered virus, and soluble MIC levels measured in culture supernatants by enzyme-linked immunosorbent assay at different time points. A-B, RV1B and RV16 increased soluble MICA (A) and soluble MICB (B) in BEAS-2B culture supernatants. C-D, RV1B increased levels of soluble MICA and MICB in a dose-responsive manner. Data are means ± SEMs of three to eight experiments. An asterisk (*) over columns represent comparisons with medium alone. *P < .05; **P < .01; ***P* < .001. MIC = major histocompatibility complex class I-related chain; MOI = multiplicity of infection; RV = rhinovirus*.

Levels of sMICA and sMICB in supernatants from respiratory epithelial cells infected with RV1B were significantly increased (at 72 h for MICA and starting earlier, at 48 h for MICB) (Figs 1A, 1B). RV1B increased sMICA and sMICB levels in a time-dependent, dose-dependent, and replication-dependent manner (Fig 1). Similar data were found using RV16 with significant induction of sMICA observed at 48 and 72 h and sMICB at 24, 48, and 72 h (Figs 1A, 1B), indicating that the sMIC induction is RV-serotype independent. sMICB levels increased earlier and were at least 10 times higher as compared with sMICA levels in RV-infected epithelial cell supernatants.

### RV Infection Increases IL-15 Production

**Figure 2:**
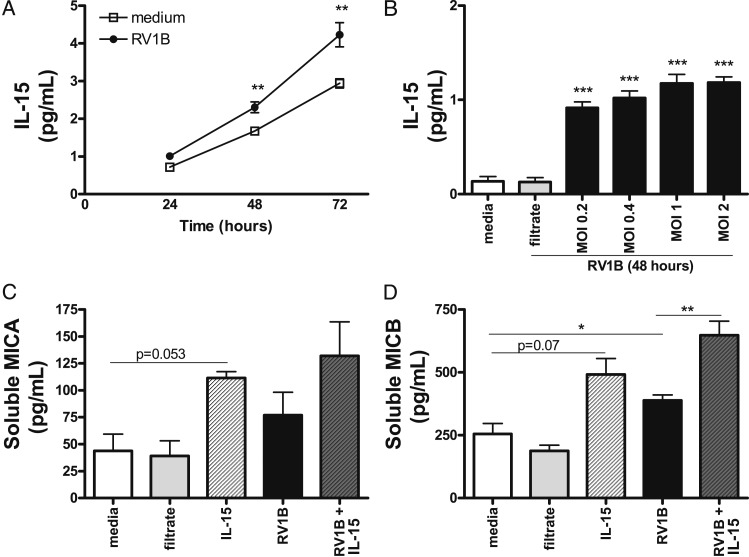
– RV infection increases IL-15 protein levels, and IL-15 increases MIC levels in bronchial respiratory epithelial cells. A, BEAS-2B cells were treated with RV1B MOI 1 or medium alone up to 72 h and IL-15 levels determined at different time points. B, BEAS-2B cells were infected with RV1B at different concentrations, filtered virus or medium alone, supernatants were harvested at 48 h, and levels of IL-15 measured. C-D, BEAS-2B cells were treated with RV1B MOI 1, filtered virus, IL-15, and RV1B + IL-15, supernatants harvested at 48 h, and levels of MICA and MICB determined. Data are means ± SEMs of three to six experiments. An asterisk (*) over columns represent comparisons with medium alone. *P < .05; **P < .01; ***P* < .001. See Figure 1 legend for expansion of abbreviations*.

RV1B and RV16 infection increased soluble IL-15 levels in supernatants from respiratory epithelial cells in a dose-dependent, replication-dependent, and time-dependent manner (Figs 2A, 2B, and data not shown). Addition of exogenous IL-15 to uninfected epithelial cells increased sMICA and sMICB level culture supernatants (*P* = .053, respectively, *P* = .079) (Figs 2C, 2D).

Addition of exogenous IL-15 to RV1B-infected epithelial cells had no effect on surface (*P* = nonsignificant, data not shown) and sMICA at 24 h postinfection (Fig 2C) (*P* = nonsignificant). However, IL-15 increased surface MICB levels at 24 h postinfection (*P* < .01 vs RV1B-infected cells, data not shown) and sMICB molecules at 48 h postinfection (RV1B vs RV1B + IL-15, *P* = .01) (Fig 2D).

### sMICA and sMICB Levels During RV Infection

**Figure 3:**
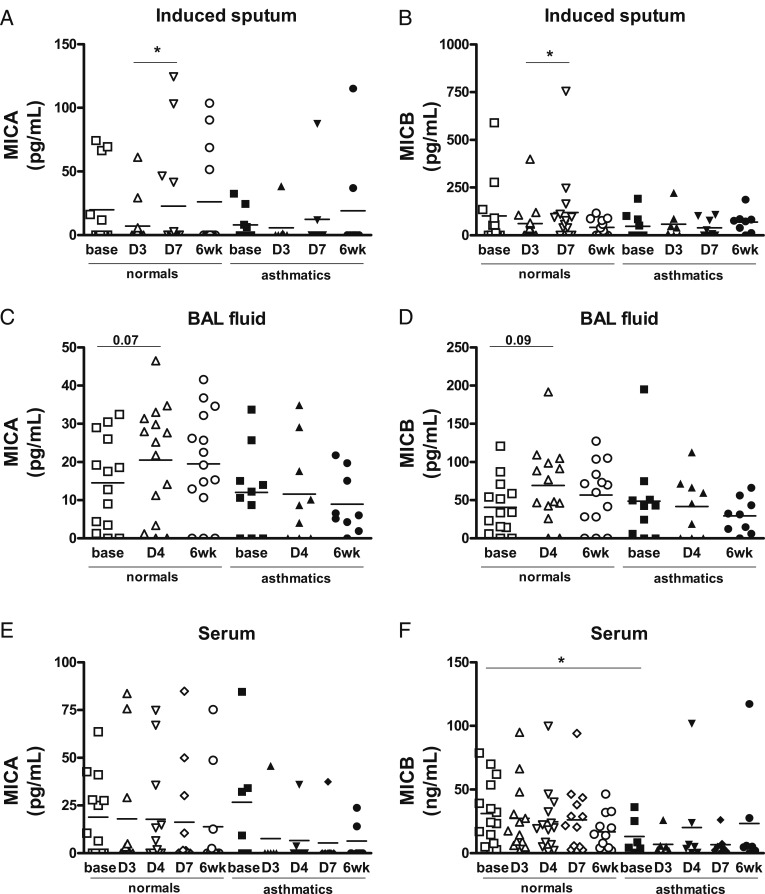
– A-F, Soluble MICA and soluble MICB levels in biologic fluids during an experimental RV16 infection in normal subjects and patients with asthma. Levels of soluble MICA and soluble MICB in normal subjects and patients with asthma at baseline, during the acute experimental RV infection, and at convalescence (6 weeks postinfection) in (A, B) induced sputum, (C, D) BAL fluid, and (E, F) serum. Data are mean and SEM. *P *< .05. See Figure 1 legend for expansion of abbreviations*.

At baseline, prior to infection, there were no significant differences between the groups in levels of sMICA and sMICB in sputum and BAL and levels of sMICA in serum (Figs 3A‐E). However, serum sMICB levels at baseline were significantly lower in atopic subjects with asthma as compared with normal subjects (Fig 3F) (*P* = .03).

There were no significant changes in sMICA and sMICB levels in sputum or BAL in the subjects with asthma following RV infection. However, in healthy subjects, levels of sMICA and sMICB increased during RV infection in sputum between days 3 and 7 (Figs 3A, 3B). There was a trend toward significantly increased levels of sMICA and sMICB in BAL at day 4 postinfection as compared with baseline (Figs 3C, 3D) (*P* = .07 and *P* = .09, respectively) in the normal subjects.

**Figure 4:**
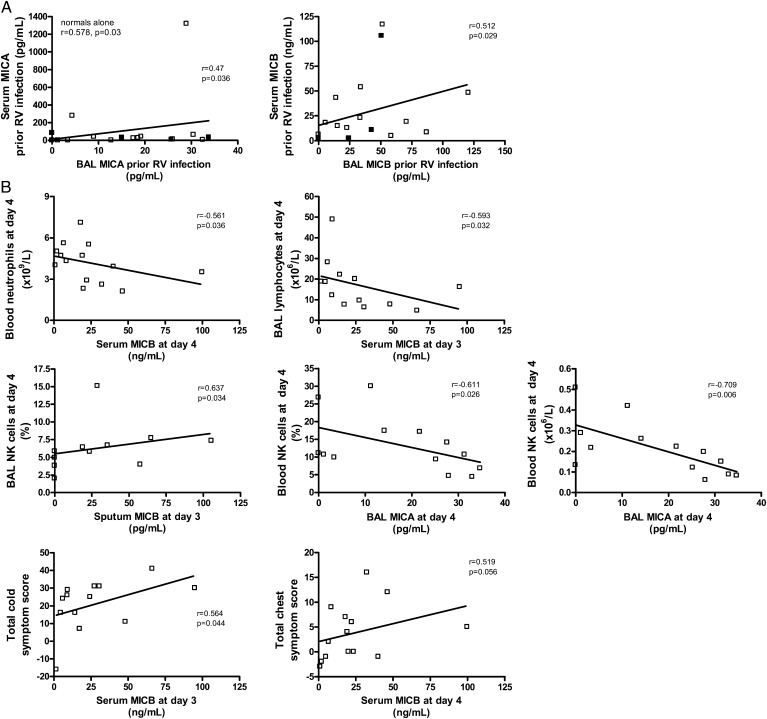
– *Relationships between soluble MIC levels to inflammatory cell type and to markers of clinical illness during an experimental RV16 infection. A, Relationships between serum and BAL MIC levels. B, Relationships between airway MIC levels and inflammatory cells and serum MIC levels and disease severity during a follow-up RV infection are shown. Data are mean and SEM. NK = natural killer. See Figure 1 legend for expansion of other abbreviations*.

sMICA levels positively correlated with sMICB levels in BAL in both healthy subjects and subjects with asthma prior to RV16 infection and at day 4 postinfection and in serum prior to RV16 infection (Table 1). Prior to RV infection there were also positive correlations between sMICA levels in serum and in BAL (all subjects: *r* = 0.47, *P* = .036; normal subjects: *r* = 0.578, *P* = .03) and between sMICB levels in serum and in BAL (all subjects: *r* = 0.512, *P* = .029) (Fig 4A).

**TABLE 1:** ] Relationship of sMICA With sMICB Levels in Biologic Fluid During an RV16 Virus Infection

Fluid	All Subjects	Normal Subjects	Subjects With Asthma
BAL			
Baseline	+0.80^a^	+0.71^a^	+0.86^a^
Day 4 pi	+0.78^a^	+0.60^a^	+0.94^a^
6 wk pi	+0.71^a^	+0.77^a^	…
Serum			
Baseline	+0.75^a^	+0.66^a^	+0.84^b^
Day 3 pi	+0.84^a^	+0.79^a^	…
Day 4 pi	+0.69^a^	+0.60^b^	+0.70^c^
Day 7 pi	+0.54^a^	+0.79^a^	…
6 wk pi	+0.65^a^	+0.72^a^	…
Sputum			
Baseline	…	…	…
Day 3 pi	0.39^c^	…	…
Day 7 pi	0.36^c^	…	…
6 wk	…	…	…

Data show the Spearman rank correlation coefficient (*r*). pi = postinfection; RV = rhinovirus; sMIC = soluble major histocompatibility complex class I-related chain.

a *P* < .01.

b *P* < .05.

c *P* < .1.

During RV infection, serum sMICB negatively correlated with blood neutrophil (*r* = −0.561, *P* = .036) and to BAL total lymphocyte numbers (*r* = −0.593, *P* = .032) in healthy subjects (Fig 4B). At the peak of RV infection in healthy subjects, sMICB levels in sputum correlated with frequency of NK cells in BAL and sMICA levels in BAL negatively correlated to the number of NK cells in blood (Fig 4B). Concerning clinical symptoms during virus infection, serum sMICB on days 3 to 4 correlated to upper (*r* = 0.564, *P* = .044) and to lower respiratory symptoms (*r* = 0.519, *P* = .056) (Fig 4B) in healthy subjects.

## Discussion

The present study is, to our knowledge, the first investigation of the potential role of MICA and MICB molecules in the pathogenesis of RV infection in healthy subjects and subjects with asthma. We report that RV infection of respiratory epithelial cells in vitro increased the levels of soluble MIC molecules MICA and MICB, and that exogenous IL-15 potentiated RV induction of sMICB molecules. Serum levels of sMICB are reduced in stable patients with asthma compared with healthy subjects and, following RV infection, sputum and BAL soluble MIC molecule levels are increased in healthy subjects, but not in patients with asthma. Interestingly, during RV infection sMICB levels in the airways correlated with increased number of BAL NK cells in healthy subjects. sMICB levels in serum correlated with clinical markers of disease severity in the healthy subjects, suggesting that these molecules may have a role in increased inflammation as a consequence of activation of immune response to RV.

The balance between inhibitory and activatory molecules/ligands on antigen-presenting cells determines whether NK and CD8 T cells are activated or inhibited to kill virus-infected cells.^22^ We previously reported that RV infection increases levels of MHC class I molecules in respiratory epithelial cells,^1^ suggesting that NK cells will be inhibited, and antiviral CD8 T cells activated, by virus antigen presentation. However, the activation of antiviral immune responses can also be nonspecific, via surface MICA/MICB molecules which bind NKG2D on NK cells and CD8 T cells.^4,23^ High levels of NKG2D stimulation via MICA/MICB can override the inhibitory signaling provided by MHC class I recognition to NK cells.^23‐25^ In the current study, we demonstrated that in vitro RV infection of respiratory epithelial cells upregulated surface expression of MICA and soluble levels of both MICA and MICB. The mechanisms of production of soluble MIC molecules have yet to be determined but it is likely that soluble molecules result from the detachment of cell-surface MIC molecules. Increased levels of sMICA were found at later time points compared with cell-surface MICA and sMICB molecules increased earlier in cell supernatants compared with MICA. A possible explanation for the discrepancy seen between surface MICB and sMICB levels is that surface MICB is induced at an earlier time point than 24 h, which was the first time point at which we measured its expression. It has been reported that surface MICA and MICB molecules are regulated differently with MICB having a much shorter half-life at the plasma membrane.^26^ Therefore, these findings support our hypothesis that soluble MIC molecules are likely to reflect cell-surface levels.

We have previously reported that RV infection increases IL-15 production in alveolar macrophages,^19^ and, therefore, we added exogenous IL-15 to determine its effect on virus-induced MICA and MICB in respiratory epithelial cells. In the presence of IL-15, RV infection significantly increased surface levels of MICB at 24 h and sMICB levels at 48 h. Therefore, the presence of a microenvironment with high IL-15 levels will favor increased expression of MICB.

To investigate the role of MIC molecules in RV infection in vivo, we measured levels of sMICA and sMICB in sputum and BAL in healthy subjects and subjects with asthma before and during an experimental RV infection. As bronchial tissue samples to directly measure surface MIC molecules were not available, we measured soluble MIC molecules in airway secretions as a surrogate marker of surface MIC expression. Following RV infection, soluble MIC molecule levels were increased in sputum and BAL in healthy subjects, but not subjects with asthma. These data suggest that in subjects with asthma there is a failure of upregulation of MIC molecules following virus infection. We have previously demonstrated that asthma is associated with IL-15 deficiency and impaired virus induction of IL-15.^19^ Therefore, impaired induction of IL-15 following virus infection may be a mechanism of reduced MIC expression in asthma.

There were positive correlations between sputum MICB levels and the percentage of BAL NK cells, and inverse correlations between BAL MICA levels and blood NK cells in healthy subjects. We have previously demonstrated inverse relationships between blood and BAL lymphocytes in experimental RV infection, presumably reflecting recruitment of lymphocytes to the lungs from the blood in response to infection. Therefore, reduced levels of MIC may be associated with reduced recruitment of NK cells and impaired antiviral immunity in asthma.

There are several limitations to our study, including the small sample size and the inclusion of subjects with mild asthma only. These factors may have contributed to failure to detect more significant differences between the groups. However, experimental RV infection studies are, by ethical reasons, restricted to small numbers of subjects with mild asthma, but provide useful hypothesis-generating human translational data that can be further investigated in controlled clinical trials performed on larger groups of patients and with more severe asthma. Furthermore, we have assumed based on our in vitro data that soluble MIC levels reflect cell-surface levels. However, further studies are required to determine whether soluble levels of MIC molecules reflect cell-surface expression in vivo and the relationships between MIC molecules and IL-15 and whether soluble MIC molecules play an active role in upregulating immune responses or simply reflect cleavage and removal of surface MIC molecules and, therefore, are a surrogate marker for cellular levels.

In conclusion, our results suggest that MIC levels in the airways are increased in response to virus infection in healthy subjects, but not subjects with asthma, and airway MICB levels correlated with NK cells at the peak of the RV infection. Impaired expression of MIC may be a mechanism of increased severity of clinical illness following virus infection in patients with asthma.

## ABBREVIATIONS

MFI: mean fluorescent intensity
MHC: major histocompatibility complex
MIC: major histocompatibility complex class I-related chain
MOI: multiplicity of infection
NK: natural killer
NKG2D: natural killer group 2 member D
RV: rhinovirus
sMIC: soluble major histocompatibility complex class I-related chain
UV: ultraviolet
